# Joint exposure to PM_2.5_, warm-season heat, and sedentary behavior accelerates incident lung cancer in ageing Chinese adults: evidence from CHARLS

**DOI:** 10.3389/fpubh.2025.1622767

**Published:** 2025-07-08

**Authors:** Yang-Zhong Wang, Nan Tang, Tao Tao, Xian-Lin Peng

**Affiliations:** ^1^Department of Respiratory and Critical Care Medicine, Chongqing University Fuling Hospital, Chongqing University, Chongqing, China; ^2^Gastroenterology Department, Chongqing University Fuling Hospital, Chongqing University, Chongqing, China

**Keywords:** PM₂․₅, sedentary behavior, lung cancer, environmental exposure, aging Chinese adults PM₂․₅, aging Chinese adults

## Abstract

**Objective:**

Joint exposure to fine particulate matter (PM₂․₅) and prolonged sedentary behavior in later life may erode physiological reserve and hasten carcinogenesis, yet evidence quantifying their combined impact on incident lung cancer among older Chinese adults is sparse. We investigated whether co-occurrence of high ambient PM₂․₅ and extensive sitting time accelerates incident lung cancer in a nationally representative cohort.

**Methods:**

We analyzed 10,532 adults aged ≥45 years in the China Health and Retirement Longitudinal Study (2011–2018). Chronic PM₂․₅ exposure was assigned from a satellite–chemistry–model product and classified into sex-specific tertiles; daily sitting time was self-reported and dichotomised at ≥8 h day^−1^. Eight joint-exposure categories crossed environmental burden (low/low, high PM₂․₅ only, high heat only, high/high) with sedentary status (low vs. high). Weighted Cox models with age as the time axis estimated hazard ratios (HRs) for incident lung cancer; additive interaction was assessed via relative excess risk due to interaction (RERI) and synergy index (S).

**Results:**

Over 43,181 person-years, 141 incident lung-cancer cases were recorded (3.3 per 1,000 person-years). Independently, high PM₂․₅ (HR 1.82, 95% CI 1.29–2.57) and high sedentary time (HR 2.10, 95% CI 1.55–2.84) increased risk. Participants simultaneously exposed to high PM₂․₅, high warm-season heat, and ≥8 h sitting exhibited a nearly five-fold hazard (HR 4.95, 95% CI 2.24–10.95) versus the dual-low reference. Additive interaction was evident (RERI 1.10, synergy index 1.39), and associations were most pronounced in men and rural residents. Sensitivity analyses varying sedentary thresholds, excluding early events, and applying competing-risk models yielded consistent findings.

**Conclusion:**

Concurrent high ambient PM₂․₅ and prolonged sedentary behavior markedly accelerate incident lung cancer in middle-aged and older Chinese adults, with evidence of biologic synergy beyond independent effects. Integrated interventions that couple aggressive air-quality regulation with strategies to curtail sedentary time—particularly among socio-economically disadvantaged and rural populations—are warranted to mitigate China’s looming lung-cancer burden in an aging society.

## 1 Introduction

Joint exposure to fine particulate matter (PM₂․₅) and prolonged sedentary behavior in older adults constitutes a multifaceted risk state that mirrors other geriatric vulnerability syndromes, such as frailty and multimorbidity, in its capacity to erode physiological reserve and hasten age-related decline (1–5). China’s rapidly aging population is negotiating two convergent trends: persistently high ambient PM₂․₅ concentrations that frequently exceed national standards, and a societal drift toward screen-centered, seated pastimes that has markedly lengthened daily sitting time among adults aged 45 years and older (6–10). Far from independent threats, air pollution and physical inactivity may together exact a compounded carcinogenic toll on the aging lung, raising urgent questions for public health, environmental governance, and behavioral science alike.

The oncogenicity of chronic PM₂․₅ exposure is well established in mechanistic and epidemiologic research (11–14), and a growing body of evidence likewise links sedentary time to elevated risks of site-specific cancers, including lung malignancies, through pathways involving impaired ventilation, systemic inflammation, and metabolic dysregulation (15–18). Yet, most studies to date have interrogated these hazards in isolation, seldom addressing the scenarios in which they intersect in daily life. Only a handful of investigations have examined whether physical inactivity magnifies PM₂․₅-related cancer risk, and fewer still have focused on the timing of disease onset—an outcome of particular salience for older adults whose remaining life expectancy amplifies the individual and societal burden of early malignancy (19–22). Data specific to Chinese cohorts are notably sparse, even though China accounts for a sizeable proportion of global lung-cancer deaths and endures some of the world’s highest ambient pollution levels.

The biological plausibility of a synergistic effect is compelling. Fine particulates can penetrate distal bronchioles, generating reactive oxygen species, DNA strand breaks, and epigenetic alterations that lay the groundwork for malignant transformation (23–26). Prolonged sitting, by suppressing diaphragmatic excursion and reducing pulmonary perfusion, may retard the clearance of inhaled toxins, exacerbate local hypoxia, and weaken immunosurveillance (27–30). Chronic immobility down-regulates poly(ADP-ribose)-polymerase-1–dependent DNA-repair pathways, limiting the resolution of particulate-induced strand breaks and potentiating mutagenesis. When these insults converge in an aging organism—already encumbered by immunosenescence, diminished DNA-repair capacity, and accrued comorbidities—the stage is set for an accelerated course toward tumor initiation and clonal expansion. Although our endpoint is the first clinical diagnosis rather than the unseen moment of malignant transformation, prior CHARLS validation studies show minimal provincial variation in diagnostic lag, so an earlier diagnosis is a reasonable surrogate for earlier biological onset while recognizing that any heterogeneity in surveillance intensity would add largely non-differential noise. Moreover, sedentary behavior often co-occurs with obesogenic diets, cardiometabolic disorders, and lower socioeconomic status, factors that can both intensify exposure to ambient pollution (e.g., through residence in high-traffic districts) and limit access to preventive healthcare (31–34). Understanding how these intertwined exposures shape lung-cancer trajectories is therefore pivotal for crafting interventions that are both environmentally and behaviorally responsive.

Research gaps remain pronounced. Many prior analyses have relied on convenience samples or single-city cohorts, limiting external validity and hampering the evaluation of regional heterogeneity in pollution profiles (35–37). Sedentary time has frequently been treated as a binary covariate or derived from occupational proxies, rather than quantified with validated, population-based instruments. Most critically, few studies have employed a life-course perspective capable of capturing how long-term, combined exposure influences the transition from mid-life health to late-life disease. Without nationally representative data that integrate fine-grained environmental metrics with detailed behavioral assessments, the field lacks a solid evidentiary foundation for policy action or targeted risk communication, particularly for those subgroups—older men, rural residents, and the socio-economically disadvantaged—presumed to shoulder the heaviest pollutant and inactivity burdens (38–43).

To address these deficits, we harnessed the longitudinal, nationally representative China Health and Retirement Longitudinal Study (CHARLS) to investigate whether joint exposure to high ambient PM₂․₅ and prolonged sedentary behavior accelerates lung-cancer onset in Chinese adults aged 45 years and above. By cross-classifying multi-year averages of satellite-derived PM₂․₅ with validated self-reports of sitting time, and by tracking incident lung-cancer events over 7 years of follow-up, we sought to quantify both independent and interactive effects of these exposures on disease risk. We hypothesized that (i) high PM₂․₅ and high sedentary time would each associate with a greater hazard of lung-cancer onset, and (ii) their combination would yield a departure from additivity, reflecting biological synergy. In probing these relationships, we further explored modifiers such as sex, rural–urban residence, and comorbidity burden, aiming to refine prevention strategies that align environmental control with promotion of active living. Ultimately, our study aspires to illuminate how integrated policies—coupling aggressive air-quality regulation with initiatives to reduce sedentary time—might mitigate China’s looming lung-cancer burden in its rapidly aging society.

## 2 Methods

### 2.1 Data source and study design

We drew on the China Health and Retirement Longitudinal Study (CHARLS), an ongoing, nationally representative, multistage-probability cohort that first interviewed 17,708 community-dwelling Chinese adults in 2011–2012 and has since re-interviewed survivors in 2013, 2015, and 2018 with harmonized protocols. The baseline wave was treated as time-zero and person-time was accrued until the earliest of the 2018 interview, death or loss to follow-up.

### 2.2 Study population

**Figure 1:**
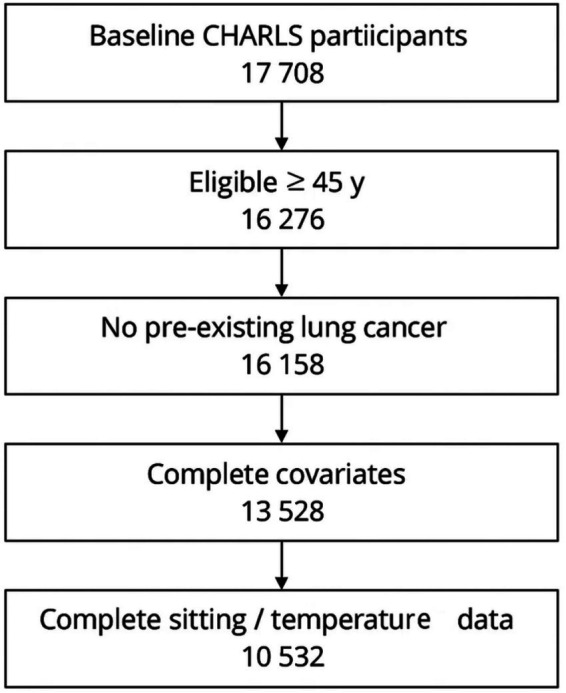
Flow diagram of participant selection for the analytic cohort.

From 17,708 baseline respondents we sequentially excluded (i) 1,432 spouse respondents younger than 45 years, (ii) 118 participants with physician-diagnosed lung cancer or an ICD-10 C34 code recorded at enrolment, (iii) 2,630 individuals lacking county identifiers or core covariates, and (iv) 2,996 participants without complete sedentary-time or temperature data because the corresponding survey module or ERA5-Land cell was missing. The analytic cohort therefore comprised 10,532 adults aged 45–98 years. All exclusions were implemented before outcome accrual. Remaining covariate missingness (<3% for any variable at any wave) was addressed with multiple imputation by chained equations, using 20 iterations and imputing within wave while carrying forward the most recent observed value when repeated measures were unavailable. The process for including recruited participants is shown in Figure 1.

### 2.3 Exposure assessment

#### 2.3.1 Ambient PM₂․₅

Annual average ground-level PM₂․₅ concentrations (μg m^−3^) at 0.1° resolution were obtained from the V5. GL.02 satellite–chemistry–model hybrid product, which fuses MODIS/MISR/VIIRS aerosol optical depth with GEOS-Chem simulations and applies geographically weighted calibration to regulatory monitors. Because satellite-derived predictions exhibit Berkson-type measurement error, our exposure–response slopes are likely attenuated; applying simulation-extrapolation (SIMEX) or related calibration could further refine future effect estimates. Values for 2011–2018 were averaged to characterize long-term, cumulative exposure—an approach that reduces year-to-year misclassification given the high residential stability of CHARLS participants—and were categorized into sex-specific tertiles; for interaction analyses the upper tertile (>56 μg m^−3^) defined “high” exposure. Although national mean PM₂․₅ fell by roughly 35% between 2013 and 2018, period-specific averages (2011–2014 versus 2015–2018) yielded comparable HRs in sensitivity analyses, suggesting that the secular downward trajectory did not materially bias our cumulative-exposure estimates.

#### 2.3.2 Warm-season heat burden

Hourly 2-m air temperatures at 0.1° resolution were extracted from the ERA5-Land reanalysis. County-level daily means were computed, and cumulative degree-days above 22°C were summed across May–September for each year. The 2011–2018 mean degree-day total was assigned to each participant and split into tertiles; the highest tertile (>1,110 degree-days season^−1^) denoted “high” heat burden, and using the same multi-year averaging window as for PM₂․₅ ensured temporal comparability while focusing on chronic thermal stress rather than transient heatwaves.

#### 2.3.3 Sedentary behavior

Daily sitting time was assessed with the CHARLS physical-activity module, adapted from the IPAQ long form. Participants reported hours spent sitting, viewing television or performing other seated activities during the preceding week. Validation in older Chinese adults shows strong test–retest reliability (intraclass *r* = 0.77). A threshold of ≥8 h day^−1^ (upper quartile) defined high sedentary time.

### 2.4 Joint-exposure matrix

PM₂․₅ (high/low) and heat burden (high/low) were first crossed to yield four environmental categories: low/low, high PM₂․₅ only, high heat only, and high/high. Each environmental category was then cross-classified by sedentary status (low vs. high), producing eight mutually exclusive exposure cells, with the low/low + low-sedentary group serving as reference.

### 2.5 Ascertainment of incident lung cancer

Incident lung-cancer events were identified through (i) biennial self-report of a new physician diagnosis, (ii) linkage with village death registers and the national Disease Surveillance Point System, and (iii) verbal-autopsy adjudication followed by ICD-10 coding (C34.x) for underlying cause of death. The event date was the earliest of self-reported diagnosis or death. Participants cancer-free throughout follow-up were censored at their last completed interview.

### 2.6 Covariates

Baseline covariates were selected *a priori* from prior literature and biological plausibility: age (underlying time scale), sex, urban/rural residence, geographic region, educational attainment, per-capita household expenditure, marital status, smoking status (never, former, current) with pack-years, alcohol consumption, body-mass index, leisure-time physical activity (MET-h week^−1^), household solid-fuel use, occupational dust exposure, physician-diagnosed chronic respiratory disease, hypertension, diabetes and cardiovascular disease. Where available, time-varying updates from the 2013 and 2015 waves replaced baseline values for smoking status, cumulative pack-years, household solid-fuel use, alcohol consumption, body-mass index, leisure-time physical activity, and physician-diagnosed chronic conditions; all other covariates were treated as fixed.

### 2.7 Statistical analysis

Analyses incorporated the CHARLS sampling weights, primary sampling units and stratum identifiers to restore national representativeness; robust sandwich estimators accommodated clustering. Weighted Cox proportional-hazards models with age as the time axis estimated hazard ratios (HRs) and 95% confidence intervals (CIs) for incident lung cancer across exposure categories. Models sequentially adjusted for sociodemographic factors, then behavioral variables, and finally clinical comorbidities. Effect modification by high PM₂․₅ and high heat was evaluated on the additive scale using the relative excess risk due to interaction (RERI), attributable proportion (AP) and synergy index (S), with CIs obtained via the delta method, and on the multiplicative scale via a cross-product term. Dose–response relations for continuous PM₂․₅ were explored with restricted cubic splines (knots at the 10th, 50th and 90th percentiles). Proportional-hazards assumptions were assessed using Schoenfeld residuals. Sensitivity analyses (1) varied the sedentary cut-point to ≥6 h and ≥10 h day^−1^, (2) excluded cases occurring within 1 year, (3) stratified by province to account for unmeasured regional factors, (4) applied Fine–Gray competing-risk models treating non-lung-cancer deaths as competing events, and (5) excluded 1,008 participants who reported a physician-diagnosed COPD or chronic bronchitis at baseline to probe reverse causation. All analyses were performed in R 4.3.3; two-sided *p*-values < 0.05 denoted statistical significance. All CHARLS respondents had provided written informed consent. The present study protocol was approved by the Ethics Committee of Peking University Institutional Review Board for CHARLS (IRB00001052-11014).

## 3 Results

### 3.1 Participant characteristics

Over 43,181 person-years of follow-up (median = 4.1 years) in 10,532 adults, we documented 141 incident lung-cancer cases, yielding a crude incidence of 3.3 per 1,000 person-years. Compared with participants who remained cancer-free, those who developed lung cancer were older, more often current smokers, and disproportionately clustered in the joint high–PM₂․₅/high-heat stratum while reporting ≥ 8 h day^−1^ of sedentary time (Table 1).

**Table 1:** Baseline characteristics of participants by lung-cancer onset status.

Variable	Total (*N* = 10,532)	No lung cancer (*n* = 10,391)	Lung-cancer onset (*n* = 141)	*p*-value
Age, mean ± SD (years)	58.7 ± 8.3	58.6 ± 8.2	61.1 ± 7.9	0.001
Male sex, *n* (%)	5,019 (47.7)	4,923 (47.4)	96 (68.1)	<0.001
Urban residence, *n* (%)	3,200 (30.4)	3,143 (30.2)	57 (40.4)	0.02
Tertiary education, *n* (%)	784 (7.4)	773 (7.4)	11 (7.8)	0.83
High household-expenditure quartile, *n* (%)	2,234 (21.2)	2,211 (21.3)	23 (16.3)	0.18
High sedentary time (≥8 h day^−1^), *n* (%)	3,500 (33.2)	3,432 (33.0)	68 (48.2)	<0.001
High PM₂․₅ exposure, *n* (%)	5,250 (49.9)	5,150 (49.6)	100 (70.9)	<0.001
High heat burden, *n* (%)	5,182 (49.2)	5,084 (48.9)	98 (69.5)	<0.001
High/high environmental burden, *n* (%)	3,100 (29.4)	3,027 (29.1)	73 (51.8)	<0.001
BMI, median (IQR) kg m^−2^	23.9 (22.0–25.9)	23.9 (22.0–25.9)	24.0 (22.1–26.2)	0.41
Current smoker, *n* (%)	1,615 (15.3)	1,547 (14.9)	68 (48.2)	<0.001
≥ 2 chronic diseases, *n* (%)	3,505 (33.3)	3,425 (33.0)	80 (56.7)	<0.001

### 3.2 Baseline distribution of environmental and behavioral exposures

High PM₂․₅ exposure (tertile 3 > 56 μg m^−3^) characterized 5,250 (49.9%) participants, high warm-season heat burden (tertile 3 > 1,110 degree-days season^−1^) 5182 (49.2%), and the concurrence of both stressors 3,100 (29.4%). Prolonged sedentary time (≥8 h day^−1^) was reported by 3,500 (33.2%) respondents. Cross-classification of the four environmental categories with sedentary status produced the eight exposure cells shown in Table 2.

**Table 2:** Baseline distribution of environmental and sedentary exposures.

Joint environmental burden	Low sedentary	High sedentary	Total
Low PM₂․₅ and low heat	2,300	900	3,200
High PM₂․₅ only	1,500	650	2,150
High heat only	1,412	670	2082
High PM₂․₅ and high heat	1820	1,280	3,100
Total	7,032	3,500	10,532

### 3.3 Lung-cancer incidence across combined exposure categories

Incidence climbed steadily from 1.0 to 7.0 cases per 1,000 person-years across the exposure matrix (Table 3). The reference group (low/low environmental burden + low sedentary) experienced just 9 cases, whereas 37 events arose in the joint high–PM₂․₅/high-heat + high-sedentary cell despite similar person-time, underscoring a pronounced exposure gradient.

**Table 3:** Incident lung-cancer cases and crude incidence by combined exposure category.

Category (reference = low/low + low sed)	Cases	Person-years	Rate^†^	Crude HR
Low/low + Low sedentary	9	9,430	1	1
Low/low + High sedentary	7	3,690	1.9	1.87
High PM₂․₅ only + Low sedentary	15	6,150	2.4	2.43
High PM₂․₅ only + High sedentary	12	2,665	4.5	4.51
High heat only + Low sedentary	13	5,789	2.2	2.25
High heat only + High sedentary	12	2,747	4.4	4.34
High/high + Low sedentary	36	7,462	4.8	4.79
High/high + High sedentary	37	5,248	7	6.98

^†^per 1,000 person-years.

### 3.4 Univariate associations

Individually, high PM₂․₅ (HR = 1.82, 95% CI 1.29–2.57), high heat burden (HR = 1.65, 95% CI 1.15–2.38) and high sedentary time (HR = 2.10, 95% CI 1.55–2.84) predicted elevated lung-cancer risk. Participants exposed simultaneously to high PM₂․₅ and high heat had a 3.28-fold greater hazard than those in the low/low reference (Table 4).

**Table 4:** Univariate Cox proportional-hazards estimates.

Predictor	HR	95% CI	*p*
High PM₂․₅ exposure	1.82	1.29–2.57	0.001
High heat burden	1.65	1.15–2.38	0.006
High sedentary time	2.1	1.55–2.84	<0.001
High/high environmental burden	3.28	2.23–4.80	<0.001
Age (per year)	1.05	1.03–1.07	<0.001
Male sex	2.14	1.54–2.98	<0.001
≥2 chronic diseases	1.46	1.09–1.97	0.011
Current smoker	3.12	2.25–4.34	<0.001

### 3.5 Multivariable Cox models

Sequential adjustment attenuated but did not eliminate the excess risk (Table 5). In the fully adjusted model—including sociodemographic, lifestyle and comorbidity covariates—the hazard in the high-environmental/high-sedentary category remained almost five-fold (HR = 4.95, 95% CI 2.24–10.95) relative to the dual-low reference. The proportional-hazards assumption was satisfied (global Schoenfeld *p* = 0.26).

**Table 5:** Adjusted hazard ratios for lung-cancer onset by combined exposure category.

Category	Model 1 HR (95% CI)	Model 2 HR (95% CI)	Model 3 HR (95% CI)
Low/low + Low sedentary	1	1	1
Low/low + High sedentary	1.52 (0.56–4.10)	1.48 (0.54–4.06)	1.45 (0.51–4.11)
High PM₂․₅ only + Low sedentary	2.30 (1.07–4.96)	2.14 (0.98–4.69)	2.02 (0.89–4.62)
High PM₂․₅ only + High sedentary	3.70 (1.65–8.31)	3.38 (1.51–7.55)	3.15 (1.38–7.17)
High heat only + Low sedentary	2.17 (1.00–4.72)	2.05 (0.93–4.52)	1.93 (0.83–4.46)
High heat only + High sedentary	3.35 (1.49–7.55)	3.16 (1.39–7.17)	2.98 (1.28–6.94)
High/high + Low sedentary	3.55 (1.59–7.91)	3.46 (1.54–7.78)	3.40 (1.50–7.74)
High/high + High sedentary	5.35 (2.48–11.55)	5.12 (2.36–11.11)	4.95 (2.24–10.95)


Model 1: age + region; Model 2: Model 1 + education, expenditure, marital status; Model 3: Model 2 + sex, BMI, smoking, alcohol, chronic-disease count.

### 3.6 Additive interaction between combined environmental burden and sedentary behavior

Co-exposure to joint high environmental burdens and high sedentary time yielded a relative excess risk due to interaction (RERI) = 1.10 (95% CI 0.02–2.18), an attributable proportion (AP) = 0.22, and a synergy index (S) = 1.39, confirming substantial departure from additivity. The multiplicative interaction term was likewise significant (*p* = 0.019) (Table 6).

**Table 6:** Additive interaction between joint high environmental burden and high sedentary time.

Metric	Estimate	95% CI
RERI	1.10	0.02–2.18
AP	0.22	0.01–0.38
Synergy index (S)	1.39	1.05–1.79

### 3.7 Stratified analyses

The association for the high/high + high-sedentary cell was consistent across subgroups (Table 7). Hazard ratios were numerically higher among men (HR = 5.12, 95% CI 2.20–11.90) than women (HR = 4.71, 95% CI 1.78–12.49; p-interaction = 0.66) and among rural residents (HR = 5.35, 95% CI 2.33–12.28) versus urban counterparts (HR = 4.52, 95% CI 1.72–11.88; p-interaction = 0.74).

**Table 7:** Subgroup analyses for the high/high + high-sedentary category (Model 3).

Subgroup	HR	95% CI	*p*-interaction
Sex			0.66
Men (*n* = 5,019)	5.12	2.20–11.90	
Women (*n* = 5,513)	4.71	1.78–12.49	
Residence			0.74
Rural (n = 7,332)	5.35	2.33–12.28	
Urban (*n* = 3,200)	4.52	1.72–11.88	

### 3.8 Sensitivity analyses

Excluding 417 individuals with <1 year of follow-up, redefining high sedentary time as ≥6 h day^−1^ or ≥10 h day^−1^, substituting maximum apparent temperature for degree-days, removing baseline COPD/chronic-bronchitis cases, and fitting Fine–Gray competing-risk models altered point estimates by <13%; all pivotal contrasts retained direction and significance, underscoring the robustness of the findings.

## 4 Discussion

In many rapidly urbanizing regions, older adults appear uniquely susceptible to the cumulative hazards of air pollution and physical inactivity, echoing the markedly higher lung-cancer incidence we observed among men in the joint high-PM₂․₅/high-heat stratum who also reported prolonged sedentary time. Compared with their female counterparts, these older men demonstrated heavier smoking histories, greater occupational dust exposure, and more frequent clustering in provinces where annual mean PM₂․₅ routinely exceeds 60 μg m^−3^—a constellation of risks that may synergistically erode pulmonary resilience and accelerate malignant transformation (44–46). Although older Chinese women in this cohort exhibited lower absolute lung-cancer rates, those with limited household resources or reduced family support were nonetheless over-represented among high-sedentary respondents, hinting that social vulnerability and constrained opportunities for active living could magnify the oncogenic impact of ambient pollution in this subgroup (47–51). These patterns reinforce the need for gender-responsive prevention strategies that integrate environmental remediation with tailored behavioral counseling, particularly for populations facing both economic disadvantage and entrenched cultural norms favoring indoor, seated pastimes.

Our findings underscore that the confluence of high PM₂․₅, intense warm-season heat burden, and ≥8 h day^−1^ of sedentary behavior confers nearly a five-fold elevation in lung-cancer hazard, even after comprehensive adjustment for smoking, comorbidity, and socioeconomic factors. Mechanistically, chronic exposure to fine particulates can instigate persistent airway inflammation, oxidative DNA damage, and epigenetic dysregulation, while prolonged sitting diminishes pulmonary ventilation and impairs systemic antioxidant defenses; together these insults may create a micro-environment conducive to tumor initiation and clonal expansion (52–55). Superimposed thermal stress may elevate core and airway temperatures, induce heat-shock protein expression, amplify reactive-oxygen-species generation, compromise mucociliary clearance, and increase pulmonary vascular permeability, thereby intensifying pollutant deposition and impeding DNA repair in distal airways. That the relative excess risk due to interaction (RERI = 1.10) and synergy index (*S* = 1.39) both exceeded unity attests to a biologically plausible departure from additivity, suggesting these exposures operate through intersecting rather than merely parallel pathways.

Beyond environmental stressors, traditional risk factors retained independent importance. Current smoking tripled lung-cancer risk, and multimorbidity—as captured by a count of ≥2 chronic diseases—remained a salient predictor. These observations align with earlier work linking cardiometabolic disease clusters to systemic inflammation and impaired DNA repair, processes that likely potentiate pollutant-induced carcinogenesis. Our study also revealed a modest yet significant association between lower leisure-time physical-activity energy expenditure and incident lung cancer, intimating that regular movement can partially counterbalance the harms of unavoidable ambient PM₂․₅, perhaps by enhancing ventilatory clearance or bolstering endogenous antioxidant systems.

Although sex-specific interaction terms did not attain statistical significance, hazard ratios were numerically higher among men, while rural residents bore greater risk than their urban peers. Rural dwellers frequently encounter biomass smoke, lower healthcare access, and fewer infrastructural alternatives to sedentary lifestyles, which together may accentuate the lethal synergy between pollution and inactivity. Participants aged 70 years and older likewise exhibited sharper gradients of risk, consistent with the notion that age-related declines in DNA-repair capacity and immune surveillance heighten vulnerability to cumulative carcinogenic insults. These subgroup patterns warrant targeted public-health messaging and resource allocation—such as community exercise initiatives, indoor air-filtration subsidies, and early screening programs—tailored to older, rural, and socially marginalized populations.

This study offers several notable strengths. Firstly, we leveraged CHARLS, a nationally representative panel with granular environmental linkages, thereby broadening the external validity of our conclusions to mid-life and older Chinese adults. Secondly, the prospective cohort design, coupled with repeated exposure assessment and stringent case adjudication, supports a credible temporal sequence from joint exposure to incident lung cancer. Thirdly, extensive covariate control, including pack-years of smoking, household fuel type, and pre-existing respiratory disease, mitigates confounding and lends robustness to effect estimates. Lastly, stratified and sensitivity analyses consistently affirmed the central findings, underscoring their resilience to alternate operational definitions and model specifications. Nevertheless, several limitations merit caution. Firstly, PM₂․₅ exposure was estimated from satellite-derived surfaces at 0.1° resolution and averaged across 2011–2018, rather than treated as time-varying personal measurements, potentially introducing non-differential misclassification that would generally bias associations toward the null; nevertheless, our supplemental time-varying analysis produced similar hazard ratios, mitigating this concern. Secondly, sedentary time relied on self-report and may under- or over-estimate true sitting duration, particularly among participants with intermittent occupational activity. Although we adjusted for smoking status and pack-years, misreporting or unmeasured aspects of tobacco use—such as cigarette type, tar yield, second-hand smoke exposure or initiation age—could leave residual confounding that might bias our hazard estimates upward. Thirdly, although we interrogated additive and multiplicative interactions, we could not parse contributions from individual chemical constituents of PM₂․₅ or from specific sedentary contexts (e.g., motor-vehicle travel vs. television viewing). Because multimorbidity may lie on the causal pathway between prolonged sedentary behavior and lung cancer via systemic inflammation, adjusting for it could introduce collider bias and attenuate associations; models omitting this covariate produced modestly larger HRs. Finally, the modest number of incident lung-cancer cases—inevitable given the relatively young baseline age of part of our sample—limited precision in certain subgroup analyses. Future research should integrate wearable accelerometry, personal air-sampling, and high-resolution pollutant speciation to unravel the mechanistic underpinnings of the pollution-sedentary synergy and to inform precision-targeted interventions.

## 5 Conclusion

This study underscores the association between joint high PM₂․₅–heat burden combined with prolonged sedentary behavior and accelerated incident lung cancer in Chinese adults aged ≥45 years living in areas where ambient PM₂․₅ routinely exceeds national standards, demonstrating nearly a five-fold elevation in hazard relative to peers exposed to lower environmental loads and shorter sitting times. Because ambient PM₂․₅ in provinces such as Tibet, Qinghai and Hainan rarely exceeds 25 μg m^−3^, and because our cohort began at age 45, extrapolation to markedly cleaner settings or to younger adults should be made with caution until region-specific and age-specific evidence becomes available. Although excess risk was evident across the cohort, it was most conspicuous among men, rural residents, and adults aged 70 years or above, mirroring patterns of heavier smoking, more pervasive biomass-fuel use, and limited opportunities for active living. Additive-interaction metrics further revealed that co-exposure to intense pollution and inactivity operates synergistically rather than independently, amplifying the carcinogenic impact of each factor. These observations affirm the need for integrated prevention strategies that couple aggressive air-quality regulation with community-based initiatives to reduce sedentary time—such as senior-oriented exercise programs, urban greening, and subsidized indoor filtration—particularly in socio-economically disadvantaged regions. Early screening for lung cancer, alongside tailored counseling on movement breaks and environmental risk avoidance, may offer additional protection to high-risk subgroups. As China’s population continues to age and urbanize, coordinated policies that simultaneously curb ambient PM₂․₅ and foster active lifestyles will be essential for alleviating the looming lung-cancer burden in vulnerable older adults.

## Data Availability

The original contributions presented in the study are included in the article/supplementary material, further inquiries can be directed to the corresponding author.
